# Pheromone expression reveals putative mechanism of unisexuality in a saprobic ascomycete fungus

**DOI:** 10.1371/journal.pone.0192517

**Published:** 2018-03-05

**Authors:** Andi M. Wilson, Magriet A. van der Nest, P. Markus Wilken, Michael J. Wingfield, Brenda D. Wingfield

**Affiliations:** Department of Genetics, Forestry and Agricultural Biotechnology Institute, University of Pretoria, Pretoria, South Africa; Soonchunhyang University, REPUBLIC OF KOREA

## Abstract

Homothallism (self-fertility) describes a wide variety of sexual strategies that enable a fungus to reproduce in the absence of a mating partner. Unisexual reproduction, a form of homothallism, is a process whereby a fungus can progress through sexual reproduction in the absence of mating genes previously considered essential for self-fertility. In this study, we consider the molecular mechanisms that allow for this unique sexual behaviour in the saprotrophic ascomycete; *Huntiella moniliformis*. These molecular mechanisms are also compared to the underlying mechanisms that control sex in *Huntiella omanensis*, a closely related, but self-sterile, species. The main finding was that *H*. *omanensis* displayed mating-type dependent expression of the **a**- and **α**-pheromones. This was in contrast to *H*. *moniliformis* where both pheromones were co-expressed during vegetative growth and sexual development. Furthermore, *H*. *moniliformis* also expressed the receptors of both pheromones. Consequently, this fungus is likely able to recognize and respond to the endogenously produced pheromones, allowing for self-fertility in the absence of other key mating genes. Overall, these results are concomitant with those reported for other unisexual species, but represent the first detailed study considering the unisexual behaviour of a filamentous fungus.

## Introduction

Sexual reproduction in fungi commonly requires an interaction between strains that carry different genetic information at the mating type locus [1]. This region, known as the *MAT1* locus in ascomycetes, harbours genes that represent either the *MAT1-1* or the *MAT1-2* idiomorph [2]. The *MAT1-1-1* gene defines the *MAT1-1* idiomorph, but can co-exist at this locus with other genes such as *MAT1-1-2* and *MAT1-1-3*. Similarly, the *MAT1-2* idiomorph is defined by the *MAT1-2-1* gene, though other genes can also be present [2]. These genes typically encode proteins with DNA binding domains and are known to regulate the global gene expression patterns associated with sexual reproduction [3].

While isolates that possess either the *MAT1-1* or *MAT1-2* idiomorph are termed heterothallic and are self-sterile, other fungi are able to sexually reproduce even in the absence of a partner [4]. This sexual strategy, homothallism, enables self-fertility and is most commonly achieved by expressing both the *MAT1-1-1* and *MAT1-2-1* genes in a single cell, as seen in *Gibberella zeae* [5] and *Aspergillus nidulans* [6]. Other molecular mechanisms enabling self-fertility have also evolved, including unisexuality [7, 8]. Unisexual reproduction occurs in individuals that would typically be described as heterothallic but are able to progress through the sexual cycle in the absence of a mating partner. These individuals possess genes only associated with one of the two *MAT* idiomorphs and thus undergo sexual reproduction in the absence of genes that are usually considered essential for sex. To date, unisexuality has been described in only five species, including the human pathogens, *Cryptococcus neoformans* and *Candida albicans* [9–13].

Fungi typically depend on protein-based sexual pheromones to attract potential mating partners and as such, pheromones are usually expressed in a mating type-dependent manner [14, 15]. Despite their obvious role in the heterothallic system, pheromones are also expressed in many homothallic species, including *A*. *nidulans* [16], *Sordaria macrospora* [17] and *G*. *zeae* [18]. This has been illustrated where the *G*. *zeae* **α**-factor pheromone has been knocked out and actually enhances facultative outcrossing in female isolates. [18] Likewise, self-fertility is abolished in strains of *S*. *macrospora* where the pheromone receptors have been deleted [19]. This indicates that these molecules influence sexual reproduction in a multitude of mechanisms that include, but are not limited to, partner attraction.

Pheromone expression has been intimately linked to the unisexual abilities of both *Ca*. *albicans* and *Cr*. *neoformans* [9, 11]. Unisexual reproduction in *Ca*. *albicans* relies on the inactivation of the *Bar1* protease, an enzyme usually required for the proteolytic breakdown of the endogenously produced **α**-factor pheromone. Upon *Bar1* inactivation, the pheromone is not broken down and the cell thus recognizes it as a signal that a compatible mating partner is present. This leads to self-mating of **a** cells which is dependent on the activation of the **α**-factor receptor [9]. In contrast, **α** cells of *Cr*. *neoformans* do not produce endogenous **a**-factor pheromone, but rely on the presence of a few or distant **a** cells to produce the pheromone which in turn enhances the fusion of **α** cells and enables same sex mating [11].

The primary subject of this study, *Huntiella moniliformis*, is a haploid filamentous ascomycete that reproduces unisexually [13]. This is despite the fact that other species in the genus, including *H*. *omanensis*, are heterothallic and require a partner for mating [13]. Since very little is known regarding the genes involved in sexual reproduction in these species, having two closely related species that undergo such vastly different sexual strategies provides an opportunity to further explore this complicated process. Furthermore, no research has been conducted on the unisexual pathway in any filamentous fungus, despite a very in depth understanding of the pathway in *Ca*. *albicans* [9, 20–22] and *Cr*. *neoformans*, both of which are yeasts [11, 23–28].

The main objective of this study was to elucidate the underlying molecular pathway associated with unisexual reproduction in *H*. *moniliformis*. Using a comparative transcriptomics approach, we sought to identify differences in gene expression between the vegetative and sexual stages of growth in *H*. *omanensis* and *H*. *moniliformis*. Ultimately, this comparison revealed key gene expression differences between heterothallic and unisexual species that we believe contribute, at least partly, to the unique sexual behaviour seen in *H*. *moniliformis*.

## Methods and materials

### Cultures

Isolates of *H*. *moniliformis* and *H*. *omanensis* previously used in the study of Wilson *et al*. [13] were used. For *H*. *moniliformis*, two distinct forms of isolate CMW36919 (CBS 144008) were used. One of these was sexually-competent, producing abundant ascomata and ascospores, while the other maintained a strictly vegetative growth form. For *H*. *omanensis*, three different isolates were used. Isolate CMW11056 (CBS 118113) is a mixed mating-type culture (MAT1xMAT2) representing the sexually reproducing isolate type. The vegetative isolates (CMW44450, CBS 143823 and CMW44442, CBS 143822), represent MAT1 and MAT2 isolates, respectively. The two vegetative cultures were isolated as single ascospore progeny from the mixed mating-type culture CMW11056 [13].

Cultures were grown and maintained on 2% malt extract agar plates (20 g.L^-1^ malt extract (Biolab, Merck, South Africa) and 20 g.L^-1^ agar (Biolab, Merck, South Africa) supplemented with thiamine hydrochloride (100 mg.L^-1^, SIGMA, Steinheim, Germany) and streptomycin sulphate salt (150 mg.L^-1^, SIGMA, Steinheim, Germany). These are forthwith referred to as MEA-ST plates. The cultures were maintained at room temperature for the duration of the study. All cultures used in this study are preserved in the culture collection (CMW) of the Forestry and Agricultural Biotechnology Institute (FABI), University of Pretoria, South Africa and at the Westerdijk Fungal Biodiversity Institute, The Netherlands.

### RNA extraction and sequencing

Total RNA was extracted from 5–7 day-old cultures. This time period ensured that cultures did not reach the stationery phase, but gave enough time for sexual reproduction to take place. Nine MEA-ST plates were used per culture, allowing for three biological and three technical replicates. Mycelium was harvested from the vegetatively growing *H*. *moniliformis* isolate as well as the MAT1 and MAT2 *H*. *omanensis* isolates. A mixture of mycelium, ascomata and ascospores was harvested from the sexually reproducing *H*. *moniliformis* (unisexual) and *H*. *omanensis* (heterosexual) cultures.

The harvested tissue was flash frozen in liquid nitrogen and ground to a fine powder using a mortar and pestle. RNA extractions were performed using the RNeasy^®^ Mini Kit (Qiagen, Limburg, The Netherlands) following the manufacturer’s protocols with the following modifications: the RLC buffer was used as the extraction buffer and the optional on-column DNase 1 digestion was included. The integrity of the total RNA was assessed by 2% (w/v) agarose gel electrophoresis at 120V for 25 min and the concentration was estimated using an ND 1000 Spectrophotometer (ThermoScientific, Waltham, USA). The RNA was further subjected to quality testing using the Experion^™^ automated electrophoresis system (BioRad Laboratories, California, USA) at the ACGT Microarray Facility (University of Pretoria, South Africa). Dynabead^®^-based mRNA enrichment (ThermoFisher Scientific, Carlsbad, USA), cDNA synthesis and library preparation were performed at the Central Analytical Facilities (CAF) at the University of Stellenbosch (South Africa). The RNA sequencing was completed at the same facility using the Ion Proton Platform and PI^™^ Chip system.

### RNA-seq analysis

CLC Genomics Workbench V7.5 (CLC bio, Aarhus, Denmark) was used to filter out raw reads with a Phred quality score of below 20 (Q ≤ 0.01) and a read length of more than 300 bp. Up to two terminal ambiguous nucleotides were trimmed from the remaining reads. To remove any reads representing the rRNA fraction of the total RNA, the filtered and/or trimmed reads were mapped to a species-specific contig harbouring only the rRNA sequences using the RNA Seq (Legacy) tool in CLC Genomics Workbench. To ensure optimal mapping, a minimum length fraction of 0.5 and a minimum similarity fraction of 0.8 were used. Reads that had not mapped to the rRNA contigs were then used in further analyses.

Gene prediction and annotation was conducted on the draft genomes assemblies of *H*. *moniliformis* (accession JMSH01000000, [29]) and *H*. *omanensis* (accession JSUI00000000, [30]) using Web AUGUSTUS with the *Fusarium graminearum* gene models [31, 32]. These gene models were used because *F*. *graminearum* is the *Huntiella* species’ closest relative for which gene models are available in the AUGUSTUS library. To detect differential expression of these predicted genes, the non-rRNA reads from each isolate type were mapped onto the species-appropriate gene annotations. Read mapping was conducted using the same length and similarity fractions as for the rRNA mapping. Intra-specific gene expression level comparisons were conducted using CLC Genomics Workbench following the method proposed by Mortazavi *et al*. [33].

For the accurate comparisons of gene expression across samples, a two-step normalization of the expression values was conducted. Firstly, RPKM values were used to represent relative expression. RPKM (Reads Per Kilobase of exon model per Million mapped reads) corrects for differences in expression levels between genes of different lengths and allows for accurate intra- and inter-sample comparisons [33]. Quantile normalization (CQN) was subsequently performed on the RPKM values in order to account for the expected technical variation specifically found in RNA-Seq data [34]. Baggerley’s Z test [35] was used to test for expression differences across comparable samples, using the normalized RPKM values. To further minimize measurement errors, a corrected p-value was used to test for statistical significance. In this case, the p-value was corrected using the Benjamini-Hochberg false discovery rate (FDR) method to adjust for multiple test correction [36].

Genes displaying a 2-fold change in expression at an FDR-corrected p-value of 0.05 and lower were considered as significantly DE. When detecting expression level changes for the *MAT* genes in *H*. *omanensis*, a different approach was employed because the sexually reproducing culture is likely made up of approximately half MAT1 material and half MAT2 material. For example, if the expression of the *MAT1-1-1* gene has the same RPKM value in both the vegetative and sexually reproducing cultures, there would have been a 2-fold expression increase in the sexually reproducing isolates. This is because the MAT1 individual expressing the gene would represent only ~50% of the culture used for RNA extraction. The same was also true for the *MAT1-1-2* gene and for the *MAT1-2-1* and *MAT1-2-7* genes in MAT2 individuals. Consequently, we considered genes displaying equal RPKM values as being significantly DE.

### Functional annotation

BLAST2GO [37] was used to assign Gene Ontology (GO) terms, InterPro identities and KEGG enzyme codes to all the genes predicted using Web AUGUSTUS. BLAST2GO also assesses GO term enrichment of one set of genes with respect to another and was used to determine whether any GO terms were enriched in the highly expressed and DE gene lists with respect to the complete list of genes from the genome. This enrichment was confirmed statistically using the Fishers Exact Test (FET) in the same program, with a p-value of 0.05 or less indicating significant enrichment. REViGO was then used to condense the enriched GO terms by removing redundant descriptions using the “small, 0.5 allowed similarity” setting. The REViGO output was then visualized using Tableau V10.1.

Genes known to be involved in sexual reproduction in other ascomycete fungi were investigated for their presence and possible DE in the two *Huntiella* species (Table 1). These included the *MAT* genes already known to be present at the *MAT* loci of the two species [13], as well as other sex-related genes from *Saccharomyces cerevisiae* [38, 39] and *Aspergillus* species [40]. The **a**- and **α**-factor pheromone genes identified based on homology to previously described Ascomycete pheromone proteins [41] were also included. In order to identify these genes in the genomes of the two *Huntiella* species, gene sequences were downloaded from the NCBI Gene Database (www.ncbi.nlm.nih.gov/gene) and used as queries in local BLASTn and tBLASTx searches against the draft genome assemblies using CLC Genomics Workbench.

**Table 1:** Genes implicated in sexual reproduction in ascomycete fungi.

Gene	Function	Species	Genbank Accession Number / NCBI Gene ID	Found in H*untiella moniliformis*	Regulation in sexually reproducing Isolate	Found in *Huntiella omanensis*	Regulation in sexually reproducing Isolate
Mating Type Genes							
*MAT1-1-1*	Primary mating type gene (alpha box)	*Huntiella omanensis*	KU950304	X	N/A	√	↑
*MAT1-1-2*	Secondary mating type gene		KU950305	X	N/A	√	-
*MAT1-2-1*	Primary mating type gene (HMG box)		KU950302	√	↑	√	↑
*MAT1-2-7*	Secondary mating type gene		KU950303	√ (Truncated)	-	√	-
Pheromone Signalling Pathway							
**a**-Factor Pheromone	Mating pheromone	*See text*	N/A	√	↓	√	-
**α**-Factor Pheromone	Mating pheromone			√	↑	√	↑
*PreA (ste3)*	a-Factor Pheromone Receptor	*Trichoderma gamsii*	29986161	√	-	√	-
*PreB (ste2)*	α-Factor Pheromone Receptor		29985002	√	-	√	↓
*KEX1*	Carboxypeptidase alpha-factor processor	*Saccharomyces cerevisiae*	852670	√	-	√	-
*KEX2*	Endoprotease alpha-factor processor		855483	√	-	√	-
*mpkB*	Mitogen-activated protein kinase	*Aspergillus clavatus*	4700387	√	↑	√	-
*sfaD*	G protein (Beta subunit)		4706342	√	-	√	-
*STE12*	Transcriptional activator, homeodomain protein	*Saccharomyces cerevisiae*	856484	√	-	√	-
*STE13*	Dipeptidyl aminopeptidase alpha-factor processor		854394	√	-	√	-
*STE20*	Serine/threonine protein kinase, MKKKK		856382	√	↑	√	-
*STE24*	Pheromone processor		853581	√	-	√	-
*steC*	Serine/threonine protein kinase, MKKK	*Aspergillus clavatus*	4706802	√	-	√	-
*CDC24*	Polarity establishment & maintenance and bud formation	*Saccharomyces cerevisiae*	851190	√	-	√	-

Symbols:

X: Gene was not identified in the genome assembly

√: Gene was identified in the genome assembly

↑: Gene was up-regulated in the sexually reproducing isolate

↓: Gene was down-regulated in the sexually reproducing isolate

-: Gene showed no differential expression

## Results

### RNA-seq statistics

High quality RNA (RQI > 9) was extracted from each of the cultures used in this study (S1 Table). Sequencing of the individual samples produced an average of ~19 million reads per library for the *H*. *moniliformis* samples and an average of ~21 million reads per library for the *H*. *omanensis* samples (S2 Table). The raw RNA-seq reads used for this study are available on the NCBI SRA database under the following accession number: SRP108437. Quality filtering and trimming of the sequence reads retained 99.9% and 99.7% of the *H*. *moniliformis* and *H*. *omanensis* reads, respectively. Once reads mapping to the rRNA contigs of the two species had been discarded, an average of more than 14 million reads per library remained for use in the subsequent analyses (S2 Table).

Of the remaining non-rRNA reads, an average of 71% and 62% mapped to the respective *H*. *moniliformis* and *H*. *omanensis* genomes (S2 Table). Unmapped reads were most likely the result of sequencing errors, reads from repetitive or unassembled genome regions [42] and/or the presence of poly(A) sequences [43]. The percentage of reads mapped to the two *Huntiella* draft genomes was similar to reports from other eukaryotic RNA mapping projects: in *S*. *cerevisiae*, approximately 56% reads mapped to the assembled genome [44], 69% in *Heterobasidion* species [45], about 50% in human [46], and 60% in cattle [43].

### Detection of gene expression

**Fig 1:**
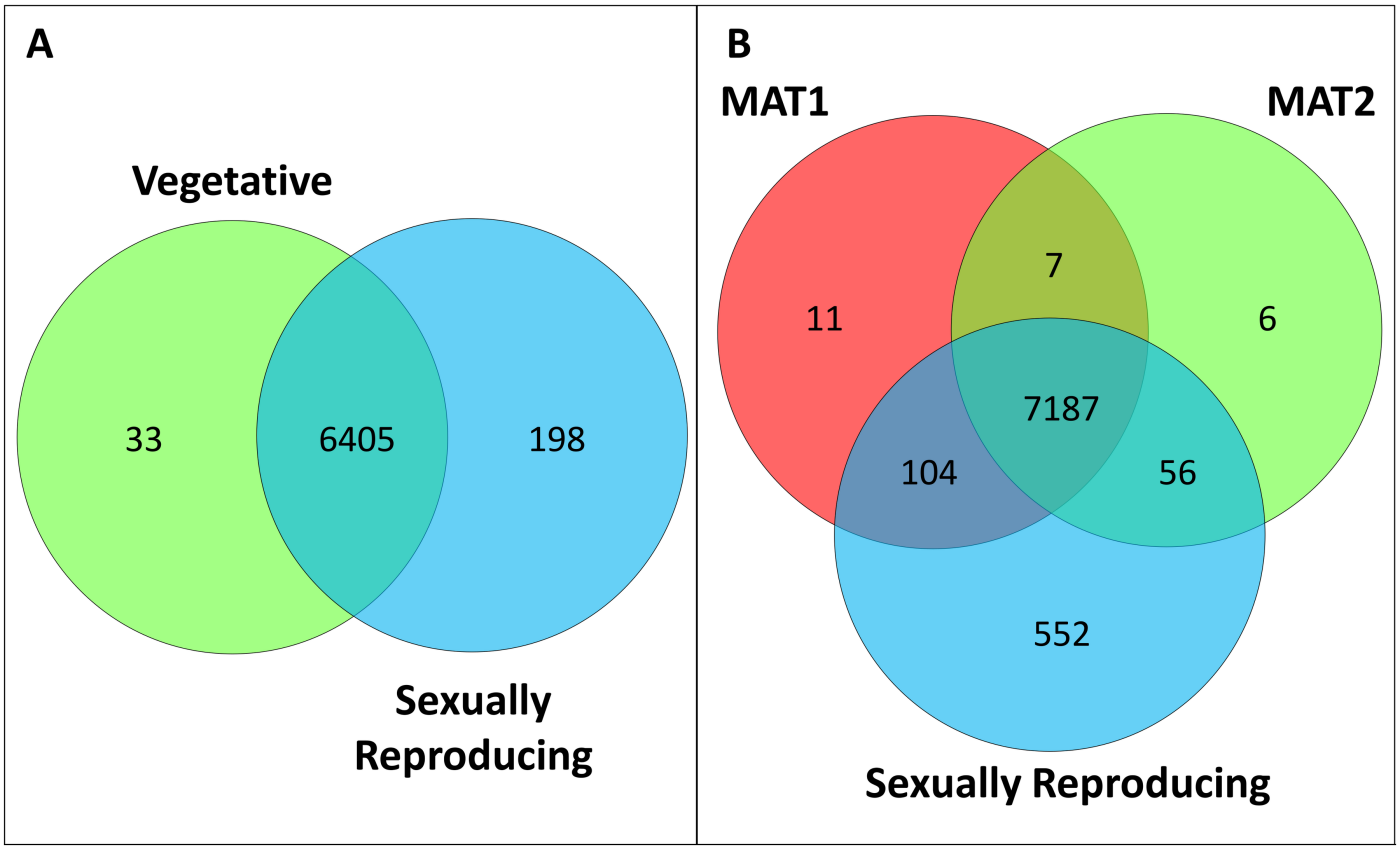
Genes expressed by vegetative and sexually reproducing isolate types of the two *Huntiella* species. **A**: The *H*. *moniliformis* draft genome has 6 864 predicted ORFs and expressed 97% of these during the course of this study. **B**: Similarly, the *H*. *omanensis* draft genome has 8 394 predicted ORFs, 94% of which were expressed.

Of the predicted 6 864 genes in the 25 Mb *H*. *moniliformis* genome, 6 636 (97%) were expressed across both isolate types. In the 31 Mb genome of *H*. *omanensis*, 7 923 (94%) of the total 8 394 predicted genes were expressed across the three isolate types. A gene was considered expressed if three or more unique gene reads mapped to its annotation [47] and a normalized RPKM value of at least 0.1 was observed [48]. The majority of the expressed genes in both species were expressed by all isolates (Fig 1).

**Fig 2:**
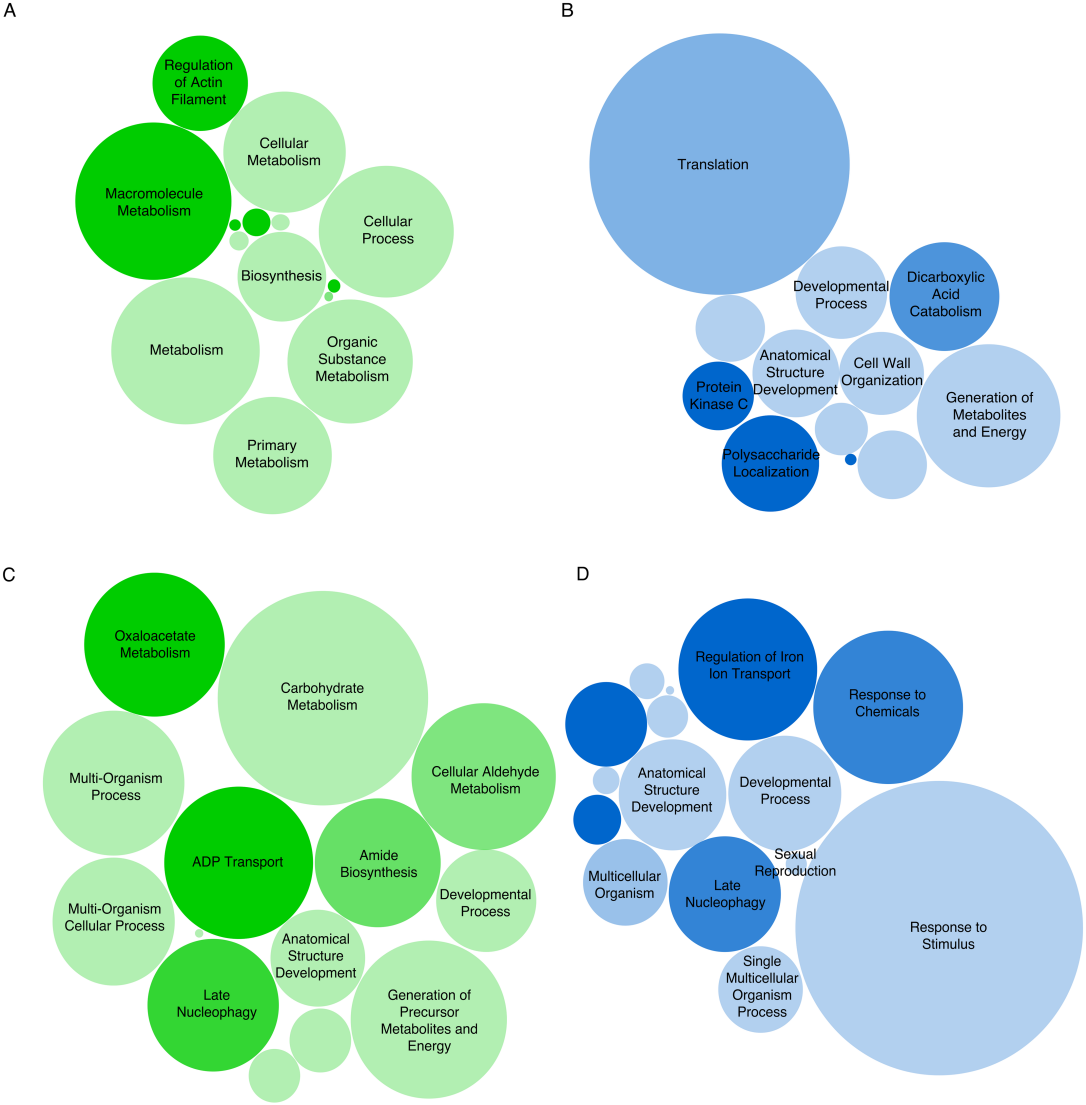
Biological process GO term enrichment in highly expressed gene sets. **A**: Vegetative and **B**: sexually reproducing cultures of *H*. *moniliformis*. **C**: Vegetative and **D**: sexually reproducing cultures of *H*. *omanensis*. The size of the circles is proportional to the relative number of GO terms associated with each process biological process. The shading intensity represents the significance of the enrichment: **A**: p-values range from 3.7 x 10^−67^ to 0.05, **B**: p-value range from 2.2 x 10^−7^ to 0.05, **C**: p-values range from 1.3 x 10^−9^ to 0.05 and **D**: p-values range from 1.7 x 10^−6^ to 0.05.

The sexually reproducing isolates of both species exhibited the highest number of uniquely expressed genes (Fig 1). Of the genes expressed in the vegetative *H*. *moniliformis* isolate, 156 were highly expressed (RPKM > 1 000, S3 Table) with 117 being unique to this isolate. The highly expressed genes were enriched for biological processes such as primary metabolism, macromolecule metabolism and biosynthesis (Fig 2, S4 Table). In the sexually reproducing isolate, 85 genes were highly expressed (S3 Table), with only 46 being unique to this isolate type. The highly expressed genes were enriched for processes such as cell wall organization, protein kinase C signalling, generation of energy and developmental processes (Fig 2, S4 Table). In the two vegetative isolates of *H*. *omanensis*, 107 genes were highly expressed, including 60 genes that were unique to the vegetative isolate type (S5 Table). These highly expressed genes were enriched for processes including glucose and carbohydrate metabolism, translational elongation and developmental processes (Fig 2, S6 Table). This is in contrast to the 74 highly expressed genes (S5 Table) in the sexually reproducing culture that included only 24 unique genes. These genes were enriched for carbohydrate catabolism, response to stress/stimulus and sexual reproduction (Fig 2, S6 Table).

### Differentially expressed genes

**Fig 3:**
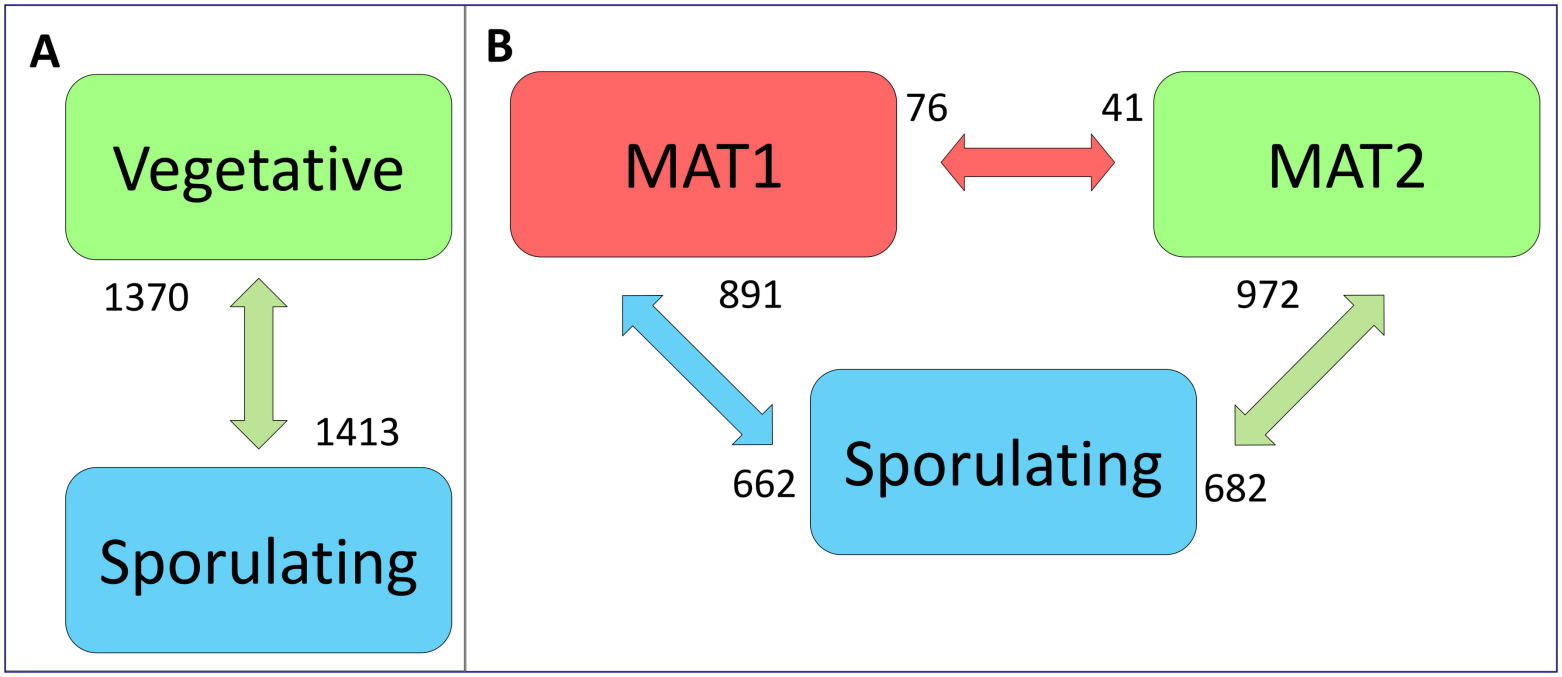
The number of genes differentially expressed in each isolate type comparison. **A**: In *H*. *moniliformis*, a similar number of genes were up-regulated in the vegetative and sexually reproducing isolates. **B**: In *H*. *omanensis*, many more genes were up-regulated in the MAT1 and MAT2 vegetative isolates than in the sexually reproducing isolate. The numbers above and below each isolate type box represent the number of genes that were up-regulated in that isolate in the particular comparison illustrated by the arrows.

Of the total gene complement of *H*. *moniliformis*, 41% of the genes were differentially expressed between vegetative and sexually reproducing isolates (Fig 3A, S7 Table). In contrast, only 24% of the total *H*. *omanensis* gene complement was differentially expressed in the same comparison (Fig 3B, S8 Table). In *H*. *moniliformis*, approximately half of the DE genes were up-regulated in the vegetative isolate and were enriched for housekeeping functions such as transcription and translation as well as RNA processing and protein transport (S9 Table). The remaining genes were up-regulated in the sexually reproducing isolates and were enriched for cell communication, signalling and signal transduction as well as carbohydrate metabolism (S9 Table). In contrast, the majority of DE genes across the *H*. *omanensis* comparisons were up-regulated in the vegetative isolates. These genes included those involved in general housekeeping processes such as carbohydrate transport and metabolism, hyphal growth and cell wall biogenesis. The remaining genes were up-regulated in the sexually reproducing isolate and included those involved in the termination of G-protein coupled receptor signalling pathways, carbon utilization and microtubule organization.

### Expression of the *MAT* genes

The *MAT* genes of the *Huntiella* species (Table 1) were identified in a previous study [13]. In *H*. *omanensis*, the primary *MAT* genes (*MAT1-1-1* and *MAT1-2-1*) were up-regulated in the sexually reproducing culture, while *MAT1-1-2* showed no evidence of differential expression. *MAT1-2-7* also showed a general pattern of up-regulation during sexual reproduction, but high intra-sample variation rendered this result insignificant. This gene was discovered using bioinformatics analyses in a previous study [13] and has no similarity to any other known proteins. Given that six other genes had already been described at the *MAT1-2* idiomorphs in a variety of other ascomycetes, this gene was named *MAT1-2-7*. This gene has recently been reported in *Knoxdaviesia* species [49]. Its association with the *MAT* locus in *H*. *omanensis* and the increase in its expression during sexual reproduction suggests that it has some unknown function during sexual reproduction. The *H*. *moniliformis MAT1-2-1* gene did not show a significant increase in expression during sexual reproduction. The truncated *H*. *moniliformis MAT1-2-7* gene was not expressed in either the vegetative or sexually reproducing cultures. This gene possesses a premature stop codon in *H*. *moniliformis* and it has been proposed that it would produce a non-functional gene product of only 48 aa in length [13].

### Expression of the pheromone response pathway

**Fig 4:**
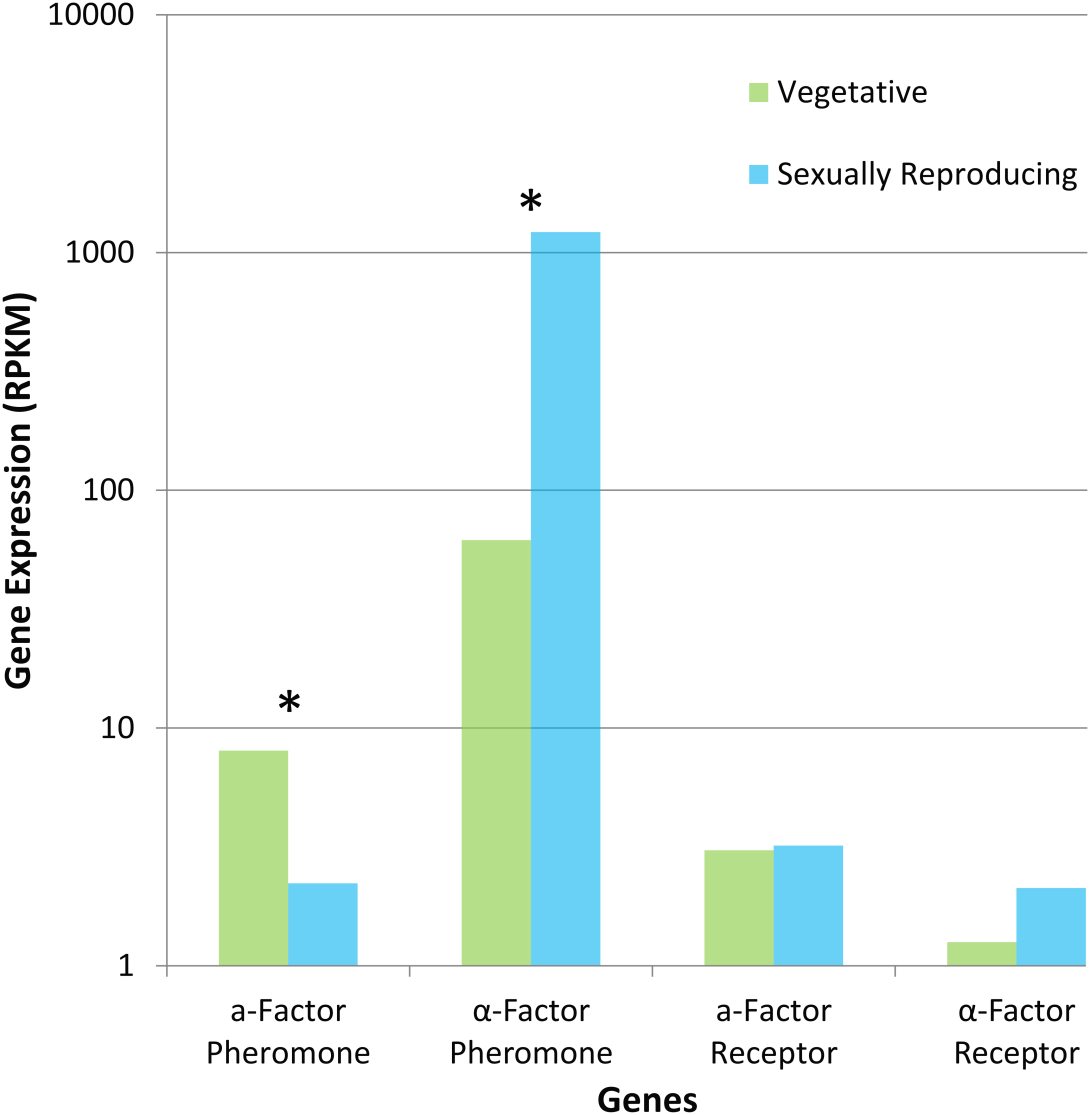
Differential expression of pheromone factor and receptor genes in *H*. *moniliformis*. Both pheromone factor genes showed differential expression, with the **a**-factor showing a significant decrease in expression in the sexually reproducing isolate while the **α**-factor showed a significant increase. The pheromone receptors showed no evidence of differential gene expression and seem to be expressed constitutively throughout the lifecycle. A logarithmic scale is used for the Y-axis. * indicates that there is a significant difference between two bars (2-fold change at FDR-corrected p-value < 0.05).

Many of the genes involved in the pheromone cascade were identified in the genomes of the two *Huntiella* species (Table 1). These included the **a**- and **α**-factor pheromones, the two **α**-pheromone processing factors, both pheromone receptors as well as several genes that play a role in the G-protein-coupled signal transduction pathway. The pheromone genes in *H*. *omanensis* were expressed in a mating-type dependent manner, with MAT1 individuals expressing the **α**-factor pheromone and MAT2 individuals expressing the **a**-factor pheromone. This was in contrast to the expression patterns of the pheromones in *H*. *moniliformis* (Fig 4), where no mating-type dependent expression was observed. The MAT2 individual expressed both the **α**-factor and **a**-factor pheromones at appreciable levels. The remaining genes were all expressed in *H*. *omanensis* and *H*. *moniliformis* in all isolate types.

There was some evidence to suggest that the pheromone receptor genes in *H*. *omanensis* exhibited mating-type dependent expression. The expression data indicated that the **α**-factor pheromone receptor was expressed solely by the MAT2 individual. The **a**-factor pheromone receptor was expressed almost exclusively by the MAT1 isolate, but was still expressed by the MAT2 isolate, albeit at much lower levels. This expression pattern did not extend to *H*. *moniliformis*. Rather, both receptor genes were expressed at a low, constitutive level in both the vegetative and sexually reproducing cultures.

The only genes that were differentially expressed in this pathway in *H*. *omanensis* were the **α**-factor pheromone (up-regulated) and its receptor (down-regulated). Again, *H*. *moniliformis* exhibited a different pattern and while the **a**-factor pheromone showed a decrease in expression in the sexually reproducing culture, the expression of the **α**-factor pheromone increased by almost 20-fold (p<0.001). Additionally, the *mpkB*, *ste20* and *ste23* genes (all involved in the signal transduction section of the response pheromone pathway) were also up-regulated in the sexually reproducing *H*. *moniliformis* culture.

## Discussion

Unisexual reproduction enables the production of sexual spores in fungi that usually require an opposite mating partner. To date, unisexuality has been observed in only five species; four ascomycetes and one basidiomycete. Although three of these species are filamentous fungi, the underlying molecular mechanisms enabling this form of self-fertility have only been characterized in the two yeast species. We compared the gene expression changes associated with unisexual reproduction in the filamentous ascomycete *H*. *moniliformis* to similar changes associated with heterothallic mating in a closely related species. The results showed that there are significant differences in the pheromone production in these two species.

A comparative transcriptomics approach revealed that changes in the expression of the pheromone precursor genes in the heterothallic *H*. *omanensis* were very similar to the general expression profiles in self-sterile model fungi such as *N*. *crassa*. Although individual isolates of *N*. *crassa* possess genes for both the **α**- and **a**-factor pheromones, they are expressed in a mating-type specific manner [50]. Thus, mat A (= MAT1) individuals express only the **α**-factor pheromone, while mat a (= MAT2) individuals express only the **a**-factor pheromone. Sexual reproduction is then initiated only in the presence of a mating partner of the opposite mating-type, despite the fact that both partners express both pheromone receptor genes [51]. Our results showed that pheromone expression in *H*. *omanensis* also follows this pattern. Interestingly, there was also some evidence of mating-type dependent receptor expression, a profile not observed in *N*. *crassa* or other heterothallic species [51].

The expression pattern of the pheromones in *H*. *moniliformis* provides a putative molecular mechanism that enables the species to reproduce unisexually. These genes exhibit neither the same mating-type dependent expression nor the differential expression seen in *H*. *omanensis*. However, they do display expression profiles similar to other homothallic species such as *S*. *macrospora* [17] and thus, both pheromones are expressed by a single isolate. It is important to recognize that species exhibiting primary homothallism, such as *S*. *macrospora*, possess both *MAT1-1* and *MAT1-2* genes, and thus, despite these genes being under mating-type control, both pheromones can be expressed. In contrast, *H*. *moniliformis* is able to achieve this expression profile, while harbouring only the *MAT1-2-1* gene.

Pheromone expression is only part of the sexual activation pathway. This is because pheromones can only elicit their intended response if recognized by the appropriate pheromone receptor. In our study, both receptors were expressed at appreciable levels by both fungal species studied. This indicates that the pheromone response pathway is being utilized. In *H*. *omanensis*, the **α**-factor receptor showed down-regulation in the sexually reproducing isolate, while the *H*. *omanensis* **a**-factor receptor as well as both receptors in *H*. *moniliformis* showed no evidence of differential expression. This result indicates that differential expression of the receptors is not important to sexual reproduction in these species and that constitutive expression is sufficient. This could also indicate that differential expression of the pheromones themselves controls the activation of sexual reproduction, irrespective of whether it is a heterothallic or homothallic interaction.

The results of this study have also provided the first experimental validation for the expression of the *MAT1-2* idiomorph-associated gene, *MAT1-2-7*. This gene was previously detected using *de novo* gene prediction software [13] and is thought to produce a full-length protein of 155 aa in *H*. *omanensis*. Its position upstream of the archetypical *MAT1-2-1* gene in MAT2 isolates coupled with its absence in the MAT1 isolates highlighted the role that it might play in mating. In the present study, *MAT1-2-7* had no detectable expression level in vegetatively growing isolates, but was shown to be expressed in sexually reproducing cultures. This expression pattern, in addition to its idiomorphic position, strongly supports a role for the *MAT1-2-7* gene in sexual reproduction. Because it is truncated in *H*. *moniliformis*, we believe it is likely that this gene plays a role in the inhibition of self-fertility.

**Fig 5:**
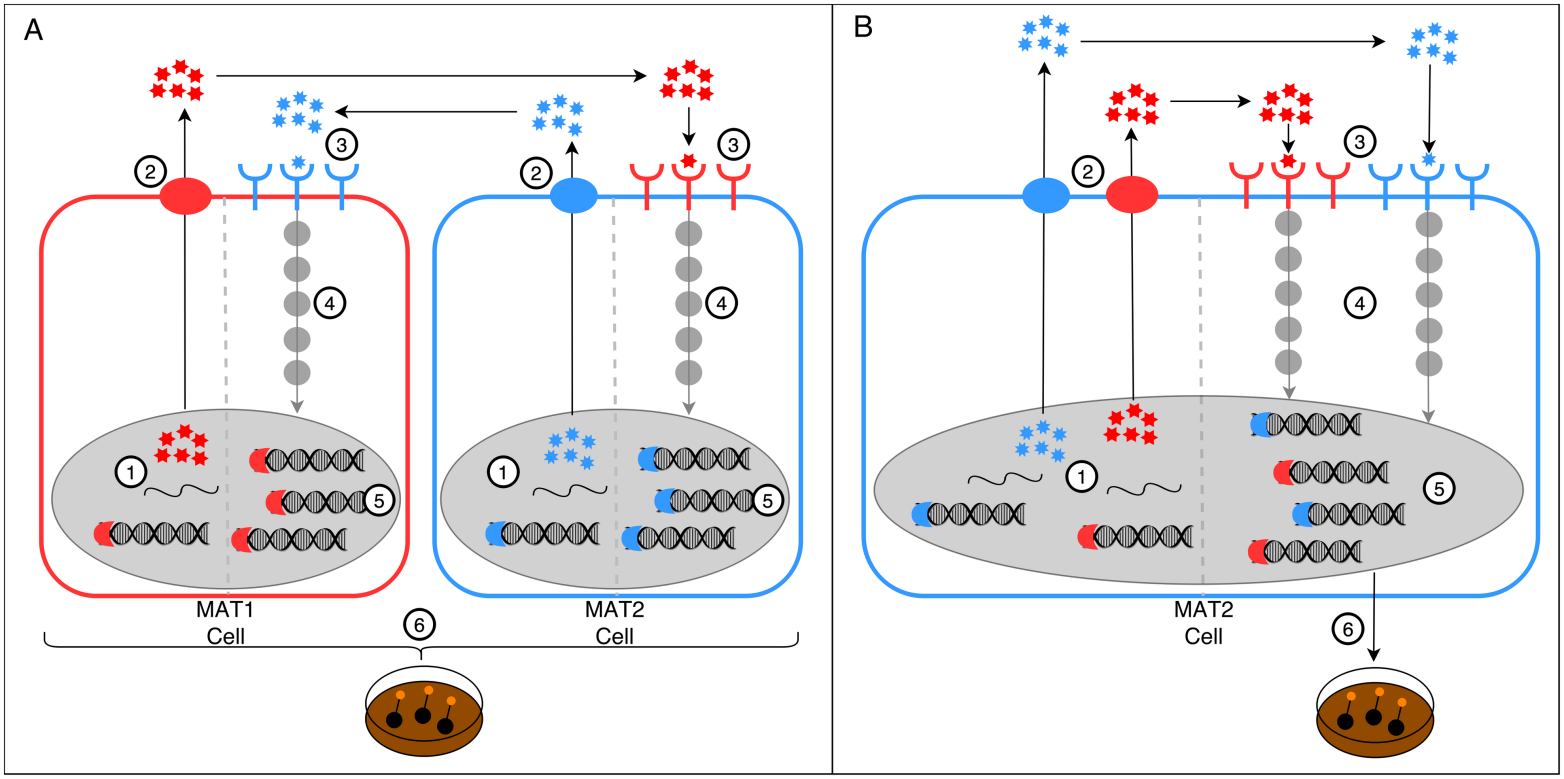
The proposed model for unisexual reproduction in *H*. *moniliformis* via the mating-type independent pheromone expression. **A**: The pheromone system in *H*. *omanensis*: MAT1 individuals express the **α**-factor pheromone and MAT2 individuals express the **a**-factor pheromone (1). These pheromones are secreted (2) and recognized by the pheromone receptors of a suitable mating partner (3). This recognition activates the pheromone transduction pathway, a MAP kinase cascade (4). Assuming other sex-favouring environmental conditions are met, this cascade alters gene expression patterns within the cells (5) and allows for the conversion of vegetative mycelia into sexually-competent tissue (6). **B**: The proposed pheromone system in *H*. *moniliformis*. The two species systems likely work in a very similar manner, except that *H*. *moniliformis* is able to express, secrete and recognize both the **α**- and **a**-factor pheromones (1, 2 & 3). Once the MAP kinase cascade has been activated (4), the genetic and physiological changes are likely very similar to those in *H*. *omanensis* (5 & 6).

*H*. *omanensis* was included in this study because it represents a typical heterothallic species. It was consequently able to provide a basis for comparison of gene expression changes that occur during a sexual interaction in *Huntiella* species. This was especially important due to the unavailability of MAT1 *H*. *moniliformis* isolates. Thus, following the model proposed for *H*. *omanensis*, expression of the **a**-factor pheromone in *H*. *moniliformis* is either directly or indirectly influenced by genes present at the *MAT1-2* idiomorph. This same model would suggest that the **α**-factor pheromone should be controlled by genes at the *MAT1-1* idiomorph. However, *H*. *moniliformis* MAT2 individuals also express this gene. Taken collectively, these results suggest that MAT2 individuals of *H*. *moniliformis* have been able to overcome the mating-type dependent expression of the pheromones that would otherwise prevent homothallic behaviour in typically heterothallic species (Fig 5).

A modification of the typical heterothallic pheromone expression pattern represents the most likely molecular mechanism that underlies unisexual reproduction in *H*. *moniliformis*. Although, the genetic elements that govern this system remain unknown, they would likely have enabled a transition from typical heterothallism to unisexual reproduction via a change in the control of pheromone expression. Comparisons between the expression patterns of the pheromone genes as well as the genic content of the *MAT1-2* loci in the two *Huntiella* species provides a basis for some speculation regarding the origin of mating-type independent pheromone expression observed in *H*. *moniliformis*. In this case, it is plausible that the pheromone expression patterns of the unisexual species could be attributed to the truncation of *MAT1-2-7* gene. This hypothesis rests on the assumption that a fully functional MAT1-2-7 protein acts as a repressor of the **α**-factor pheromone in MAT2 individuals, with its absence in MAT1 individuals allowing for expression of this pheromone. The genes of the *MAT* locus have been shown to be essential for pheromone expression in *N*. *crassa* [50], *S*. *macrospora* [52] and *C*. *neoformans* [25] and provides a precedent for the direct involvement of the MAT proteins in the pheromone system. In fact, a recent study has shown that the inactivation of the Mat2 gene in *C*. *neoformans* disables the pheromone pathway and preferentially enables unisexual behaviour over bisexual mating [24].

The hypothesis that the truncated *MAT1-2-7* gene is responsible for the unisexual behaviour of *H*. *moniliformis* is further supported by mutation studies performed on the heterothallic *P*. *anserina* [53]. Here, disruptions in a number of the *MAT* genes resulted in self-fertility. Importantly, it has been shown that the wild type mat+ genes (= *MAT1-2*) are not only required for the activation of mat+ functions, but also for the repression of *mat-* (= *MAT1-1*) fertilization functions. Thus, when there is a mutation in the mat+ gene, FPR1, the repression of fertilization is lifted and the mutant is rendered self-fertile. We suggest that this could be similar to what occurs in *H*. *omanensis*, where a full length *MAT1-2-7* gene represses the **α**-factor pheromone, rendering the MAT2 isolates self-sterile. However, in *H*. *moniliformis*, the *MAT1-2-7* gene is truncated, the repression of the **α**-factor pheromone does not occur and mating-type independent expression is possible. This in turn leads to self-activation, self-fertility and culminates in unisexual reproduction.

The results of this study suggest that the mating-type independent expression of the **a**- and **α**-factor pheromones is responsible for the unique ability of the MAT2 individuals of *H*. *moniliformis* to complete a sexual cycle in the absence of a MAT1 mating partner. As presented above, the role of pheromone expression in unisexuality has been previously investigated in both *Ca*. *albicans* and *Cr*. *neoformans* [9, 20, 23–26]. The results presented here are most similar to observations for *Ca*. *albicans*, and we hypothesize that endogenous production of both the **a**- and **α**-factor pheromones by a single MAT2 individual is sufficient to initiate the sexual cycle in *H*. *moniliformis*. We believe that this is the most probable explanation for the data presented here but acknowledge that other plausible hypotheses may also exist.

The unisexual pathway exhibited by *H*. *moniliformis* was originally assumed to be a sexual process. This was partly due to the similarity of the reproductive structures and spores found in *H*. *moniliformis* and the heterothallic *H*. *omanensis* [13]. The apparent importance of pheromone expression in the unisexual pathway of *H*. *moniliformis* further substantiates this assumption. However, it is worth noting that another spore-producing process, known as haploid fruiting, also enables spore production in single mating type isolates. Although similar to unisexuality in that way, this process is not necessarily linked to sexual reproduction or the formation of a diploid state [54–56]. It may even require different environmental conditions to those necessary for sexual reproduction [57]. Haploid fruiting can be induced in *Schizophyllum commune* when the mycelium is physically damaged [55] or when a fruiting-inducing chemical is present in the environment [54]. While this raises an alternative hypothesis for the process through which *H*. *moniliformis* produces progeny, the currently available data supports a sexual process.

This study utilized a transcriptomics approach that has some inherent limitations. The most important is that conclusions drawn from the data assume that gene expression is directly correlated with the production of a functional protein product. In addition, pheromones are often expressed as pre-pheromone proteins that require post-translational modification. Therefore, future studies will include the functional characterization of the two pheromone genes as well as the *MAT1-2-7* gene in order to enhance our understanding of the role these genes play in unisexuality. Furthermore, studies utilizing synthetic pheromones will be conducted in an attempt to initiate unisexual behaviour in *H*. *omanensis*.

## Supporting information

S1 Table Quality and concentration of total RNA extractions from vegetative and sexually reproducing cultures of *H*. *omanensis* and *H*. *moniliformis*. (XLSX)

S2 Table RNA sequencing and genome mapping statistics. (XLSX)

S3 Table Genes highly expressed in vegetative and sexually reproducing cultures of *H*. *moniliformis*. (XLSX)

S4 Table FET enriched GO terms associated with the genes highly expressed in vegetative and sexually reproducing cultures of *H*. *moniliformis*. (XLSX)

S5 Table Genes highly expressed in vegetative and sexually reproducing cultures of *H*. *omanensis*. (XLSX)

S6 Table FET enriched GO terms associated with the genes highly expressed in vegetative and sexually reproducing cultures of *H*. *omanensis*. (XLSX)

S7 Table Genes differentially expressed in vegetative and sexually reproducing cultures of *H*. *moniliformis*. (XLSX)

S8 Table Genes differentially expressed in vegetative and sexually reproducing cultures of *H*. *omanensis*. (XLSX)

S9 Table FET enriched GO terms associated with the genes up-regulated in vegetative and sexually reproducing cultures of *H*. *moniliformis*. (XLSX)

S10 Table FET enriched GO terms associated with the genes grouped in cluster A and B of the DE genes of *H*. *omanensis*. (XLSX)

## References

[1] Coppin E, Debuchy R, Arnaise S, Picard M. Mating Types and Sexual Development in Filamentous Ascomycetes. Microbiology and Molecular Biology Reviews. 1997;61(4):411–28. 9409146 10.1128/mmbr.61.4.411-428.1997 PMC232618

[2] Turgeon BG, Yoder O. Proposed Nomenclature for Mating Type Genes of Filamentous Ascomycetes. Fungal Genetics and Biology. 2000;31(1):1–5. doi: 10.1006/fgbi.2000.1227 11118130 10.1006/fgbi.2000.1227

[3] Kronstad JW, Staben C. Mating Type in Filamentous Fungi. Annual Review of Genetics. 1997;31:245–76. doi: 10.1146/annurev.genet.31.1.245 9442896 10.1146/annurev.genet.31.1.245

[4] Lin X, Heitman J. Mechanisms of Homothallism in Fungi and Transitions between Heterothallism and Homothallism In: Heitman J, Kronstad JW, Taylor JW, Casselton LA, editors. Sex in Fungi: Molecular Determination and Evolutionary Implications. Washington, D.C.: ASM Press; 2007 p. 35–57.

[5] Bowden RL, Leslie JF. Sexual Recombination in *Gibberella zeae*. Phytopathology. 1999;89(2):182–8. doi: 10.1094/PHYTO.1999.89.2.182 18944794 10.1094/PHYTO.1999.89.2.182

[6] Pontecorvo G, Roper J, Hemmons LM, MacDonald K, Bufton A. The Genetics of *Aspergillus nidulans*. Advances in Genetics. 1953; (5):141–238. 13040135 10.1016/s0065-2660(08)60408-3

[7] Roach K, Feretzaki M, Sun S, Heitman J. Unisexual Reproduction. Advances in Genetics. 2014;85:255–305. doi: 10.1016/B978-0-12-800271-1.00005-6 24880737 10.1016/B978-0-12-800271-1.00005-6 PMC9036533

[8] Wilson AM, Wilken PM, van der Nest MA, Steenkamp ET, Wingfield MJ, Wingfield BD. Homothallism: An Umbrella Term for describing Diverse Sexual Behaviours. IMA Fungus. 2015;6(1):207 doi: 10.5598/imafungus.2015.06.01.13 26203424 10.5598/imafungus.2015.06.01.13 PMC4500084

[9] Alby K, Schaefer D, Bennett RJ. Homothallic and Heterothallic Mating in the Opportunistic Pathogen *Candida albicans*. Nature. 2009;460(7257):890–3. doi: 10.1038/nature08252 19675652 10.1038/nature08252 PMC2866515

[10] Glass NL, Smith ML. Structure and Function of a Mating-Type Gene from the Homothallic Species *Neurospora africana*. Molecular and General Genetics 1994;244(4):401–9. 8078466 10.1007/BF00286692

[11] Lin X, Hull CM, Heitman J. Sexual Reproduction between Partners of the Same Mating Type in *Cryptococcus neoformans*. Nature. 2005;434(7036):1017–21. doi: 10.1038/nature03448 15846346 10.1038/nature03448

[12] Schuerg T, Gabriel R, Baecker N, Baker SE, Singer SW. *Thermoascus aurantiacus* is an Intriguing Host for the Industrial Production of Cellulases. Current Biotechnology. 2017;6(2):89–97.

[13] Wilson A, Godlonton T, van der Nest M, Wilken P, Wingfield M, Wingfield B. Unisexual Reproduction in *Huntiella moniliformis*. Fungal Genetics and Biology. 2015;80:1–9. doi: 10.1016/j.fgb.2015.04.008 25910452 10.1016/j.fgb.2015.04.008

[14] Coppin E, de Renty C, Debuchy R. The Function of the Coding Sequences for the Putative Pheromone Precursors in *Podospora anserina* is Restricted to Fertilization. Eukaryotic Cell. 2005;4(2):407–20. doi: 10.1128/EC.4.2.407-420.2005 15701803 10.1128/EC.4.2.407-420.2005 PMC549327

[15] Shen W-C, Bobrowicz P, Ebbole DJ. Isolation of Pheromone Precursor Genes of *Magnaporthe grisea*. Fungal Genetics and Biology. 1999;27(2):253–63. 10441451 10.1006/fgbi.1999.1151

[16] Paoletti M, Seymour FA, Alcocer MJ, Kaur N, Calvo AM, Archer DB, et al Mating Type and the Genetic Basis of Self-Fertility in the Model Fungus *Aspergillus nidulans*. Current Biology. 2007;17(16):1384–9. doi: 10.1016/j.cub.2007.07.012 17669651 10.1016/j.cub.2007.07.012

[17] Pöggeler S. Two Pheromone Precursor Genes are Transcriptionally Expressed in the Homothallic Ascomycete *Sordaria macrospora*. Current Genetics. 2000;37(6):403–11. 10905431 10.1007/s002940000120

[18] Lee J, Leslie JF, Bowden RL. Expression and Function of Sex Pheromones and Receptors in the Homothallic Ascomycete *Gibberella zeae*. Eukaryotic Cell. 2008;7(7):1211–21. doi: 10.1128/EC.00272-07 18503004 10.1128/EC.00272-07 PMC2446672

[19] Klix V, Nowrousian M, Ringelberg C, Loros J, Dunlap J, Pöggeler S. Functional Characterization of *MAT1-1*-Specific Mating-Type Genes in the Homothallic Ascomycete *Sordaria macrospora* provides New Insights into Essential and Nonessential Sexual Regulators. Eukaryotic Cell. 2010;9(6):894–905. doi: 10.1128/EC.00019-10 20435701 10.1128/EC.00019-10 PMC2901639

[20] Alby K, Bennett RJ. Interspecies Pheromone Signaling Promotes Biofilm Formation and Same-Sex Mating in *Candida albicans*. Proceedings of the National Academy of Sciences. 2011;108(6):2510–5. 10.1073/pnas.1017234108 PMC3038756 21262815

[21] Bennett RJ, Johnson AD. Completion of a Parasexual Cycle in *Candida albicans* by Induced Chromosome Loss in Tetraploid Strains. The EMBO Journal. 2003;22(10):2505–15. doi: 10.1093/emboj/cdg235 12743044 10.1093/emboj/cdg235 PMC155993

[22] Schaefer D, Côte P, Whiteway M, Bennett RJ. Barrier Activity in *Candida albicans* Mediates Pheromone Degradation and Promotes Mating. Eukaryotic Cell. 2007;6(6):907–18. doi: 10.1128/EC.00090-07 17416895 10.1128/EC.00090-07 PMC1951518

[23] Feretzaki M, Heitman J. Genetic Circuits that govern Bisexual and Unisexual Reproduction in *Cryptococcus neoformans*. PLoS Genetics. 2013;9(8):e1003688 doi: 10.1371/journal.pgen.1003688 23966871 10.1371/journal.pgen.1003688 PMC3744442

[24] Gyawali R, Zhao Y, Lin J, Fan Y, Xu X, Upadhyay S, et al Pheromone Independent Unisexual Development in *Cryptococcus neoformans*. PLoS Genetics. 2017;13(5):e1006772 doi: 10.1371/journal.pgen.1006772 28467481 10.1371/journal.pgen.1006772 PMC5435349

[25] Lin X, Jackson JC, Feretzaki M, Xue C, Heitman J. Transcription Factors Mat2 and Znf2 Operate Cellular Circuits Orchestrating Opposite-and Same-Sex Mating in *Cryptococcus neoformans*. PLoS Genetics. 2010;6(5):e1000953 doi: 10.1371/journal.pgen.1000953 20485569 10.1371/journal.pgen.1000953 PMC2869318

[26] Ni M, Feretzaki M, Li W, Floyd-Averette A, Mieczkowski P, Dietrich FS, et al Unisexual and Heterosexual Meiotic Reproduction Generate Aneuploidy and Phenotypic Diversity *de novo* in the Yeast *Cryptococcus neoformans*. PLoS Biology. 2013;11(9):e1001653 doi: 10.1371/journal.pbio.1001653 24058295 10.1371/journal.pbio.1001653 PMC3769227

[27] Wang P, Perfect JR, Heitman J. The G-protein β subunit GPB1 is required for Mating and Haploid Fruiting in *Cryptococcus neoformans*. Molecular and Cellular Biology. 2000;20(1):352–62. 10594037 10.1128/mcb.20.1.352-362.2000 PMC85090

[28] Wickes BL, Mayorga ME, Edman U, Edman JC. Dimorphism and Haploid Fruiting in *Cryptococcus neoformans*: Association with the α Mating Type. Proceedings of the National Academy of Sciences. 1996;93(14):7327–31. 10.1073/pnas.93.14.7327 PMC38983 8692992

[29] van der Nest MA, Bihon W, De Vos L, Naidoo K, Roodt D, Rubagotti E, et al Draft Genome Sequences of *Diplodia sapinea*, *Ceratocystis manginecans*, and *Ceratocystis moniliformis*. IMA Fungus. 2014;5(1):135–40. doi: 10.5598/imafungus.2014.05.01.13 25083413 10.5598/imafungus.2014.05.01.13 PMC4107891

[30] Van der Nest MA, Wilken PM, Naidoo K, Roodt D, Crouch JA, Demers JE, et al *Draft Genome* Sequences of *Amanita jacksonii*, *Ceratocystis albifundus*, *Fusarium circinatum*, *Huntiella omanensis*, *Leptographium procerum*, *Rutstroemia sydowiana* and *Sclerotinia echinophila*. IMA Fungus. 2014;5:473–85. 25734036 10.5598/imafungus.2014.05.02.11 PMC4329328

[31] Stanke M, Steinkamp R, Waack S, Morgenstern B. AUGUSTUS: A Web Server for Gene Finding in Eukaryotes. Nucleic Acids Research. 2004;32(2):W309–W12. 15215400 10.1093/nar/gkh379 PMC441517

[32] Stanke M, Waak S. Gene Prediction with a Hidden-Markov Model and a New Intron Submodel. Bioinformatics. 2003;19:ii215–ii25. 14534192 10.1093/bioinformatics/btg1080

[33] Mortazavi A, Williams BA, McCue K, Schaeffer L, Wold B. Mapping and Quantifying Mammalian Transcriptomes by RNA-Seq. Nature methods. 2008;5(7):621–8. doi: 10.1038/nmeth.1226 18516045 10.1038/nmeth.1226 PMC13303166

[34] Hansen KD, Irizarry RA, Zhijin W. Removing Technical Variability in RNA-Seq Data using Conditional Quantile Normalization. Biostatistics. 2012;13(2):204–16. doi: 10.1093/biostatistics/kxr054 22285995 10.1093/biostatistics/kxr054 PMC3297825

[35] Baggerly KA, Deng L, Morris JS, Aldaz CM. Differential Expression in SAGE: Accounting for Normal Between-Library Variation. Bioinformatics. 2003;19(12):1477–83. 12912827 10.1093/bioinformatics/btg173

[36] Benjamini Y, Hochberg Y. Controlling the False Discovery Rate: A Practical and Powerful Approach to Multiple Testing. Journal of the Royal Statistical Society. 1995;57(1):289–300.

[37] Conesa A, Götz S, García-Gómez JM, Terol J, Talón M, Robles M. Blast2GO: A Universal Tool for Annotation, Visualization and Analysis in Functional Genomics Research. Bioinformatics. 2005;21(18):3674–6. doi: 10.1093/bioinformatics/bti610 16081474 10.1093/bioinformatics/bti610

[38] Mitchell AP. Control of Meiotic Gene Expression in *Saccharomyces cerevisiae*. Microbiological Reviews. 1994;58(1):56–70. 8177171 10.1128/mr.58.1.56-70.1994 PMC372953

[39] Spellman PT, Sherlock G, Zhang MQ, Iyer VR, Anders K, Eisen MB, et al Comprehensive Identification of Cell Cycle—Regulated Genes of the Yeast *Saccharomyces cerevisiae* by Microarray Hybridization. Molecular Biology of the Cell. 1998;9(12):3273–97. 9843569 10.1091/mbc.9.12.3273 PMC25624

[40] Fedorova ND, Khaldi N, Joardar VS, Maiti R, Amedeo P, Anderson MJ, et al Genomic Islands in the Pathogenic Filamentous Fungus *Aspergillus fumigatus*. PLoS Genetics. 2008;4(4):e1000046 doi: 10.1371/journal.pgen.1000046 18404212 10.1371/journal.pgen.1000046 PMC2289846

[41] Martin SH, Wingfield BD, Wingfield MJ, Steenkamp ET. Causes and Consequences of Variability in Peptide Mating Pheromones of Ascomycete Fungi. Molecular Biology and Evolution. 2011;28(7):1987–2003. doi: 10.1093/molbev/msr022 21252281 10.1093/molbev/msr022

[42] Marioni JC, Mason CE, Mane SM, Stephens M, Gilad Y. RNA-Seq: An Assessment of Technical Reproducibility and Comparison with Gene Expression Arrays. Genome Research. 2008;18(9):1509–17. doi: 10.1101/gr.079558.108 18550803 10.1101/gr.079558.108 PMC2527709

[43] Huang W, Khatib H. Comparison of Transcriptomic Landscapes of Bovine Embryos using RNA-Seq. BMC Genomics. 2010;11(1):711. 21167046 10.1186/1471-2164-11-711 PMC3019235

[44] Nagalakshmi U, Wang Z, Waern K, Shou C, Raha D, Gerstein M, et al The Transcriptional Landscape of the Yeast Genome defined by RNA Sequencing. Science. 2008;320(5881):1344–9. doi: 10.1126/science.1158441 18451266 10.1126/science.1158441 PMC2951732

[45] Van der Nest MA, Olson Å, Karlsson M, Lind M, Dalman K, Brandström-Durling M, et al Gene Expression Associated with Intersterility in *Heterobasidion*. Fungal Genetics and Biology. 2014;73:104–19. doi: 10.1016/j.fgb.2014.10.008 25459536 10.1016/j.fgb.2014.10.008

[46] Sultan M, Schulz MH, Richard H, Magen A, Klingenhoff A, Scherf M, et al A Global View of Gene Activity and Alternative Splicing by Deep Sequencing of the Human Transcriptome. Science. 2008;321(5891):956–60. doi: 10.1126/science.1160342 18599741 10.1126/science.1160342

[47] Wickramasinghe S, Rincon G, Islas-Trejo A, Medrano JF. Transcriptional Profiling of Bovine Milk using RNA Sequencing. BMC Genomics. 2012;13(1):45. 22276848 10.1186/1471-2164-13-45 PMC3285075

[48] Ramsköld D, Luo S, Wang Y-C, Li R, Deng Q, Faridani OR, et al Full-Length mRNA-Seq from Single-Cell Levels of RNA and Individual Circulating Tumor Cells. Nature Biotechnology. 2012;30(8):777–82. doi: 10.1038/nbt.2282 22820318 10.1038/nbt.2282 PMC3467340

[49] Aylward J, Steenkamp ET, Dreyer LL, Roets F, Wingfield MJ, Wingfield BD. Genetic Basis for High Population Diversity in Protea-Associated *Knoxdaviesia*. Fungal Genetics and Biology. 2016;96:47–57. doi: 10.1016/j.fgb.2016.10.002 27720822 10.1016/j.fgb.2016.10.002

[50] Bobrowicz P, Pawlak R, Correa A, Bell-Pedersen D, Ebbole DJ. The *Neurospora crassa* Pheromone Precursor Genes are Regulated by the Mating Type Locus and the Circadian Clock. Molecular Microbiology. 2002;45(3):795–804. 12139624 10.1046/j.1365-2958.2002.03052.x

[51] Pöggeler S, Kück U. Identification of Transcriptionally Expressed Pheromone Receptor Genes in Filamentous Ascomycetes. Gene. 2001;280(1):9–17. 11738813 10.1016/s0378-1119(01)00786-7

[52] Pöggeler S, Nowrousian M, Ringelberg C, Loros J, Dunlap J, Kück U. Microarray and Real-Time PCR Analyses Reveal Mating Type-Dependent Gene Expression in a Homothallic Fungus. Molecular Genetics and Genomics. 2006;275(5):492–503. doi: 10.1007/s00438-006-0107-y 16482473 10.1007/s00438-006-0107-y

[53] Arnaise S, Zickler D, Le Bilcot S, Poisier C, Debuchy R. Mutations in Mating-Type Genes of the Heterothallic Fungus *Podospora anserina* lead to Self-Fertility. Genetics. 2001;159(2):545–56. 11606532 10.1093/genetics/159.2.545 PMC1461809

[54] Leonard TJ, Dick S. Chemical Induction of Haploid Fruiting Bodies in *Schizophyllum commune*. Proceedings of the National Academy of Sciences. 1968;59(3):745–51. 10.1073/pnas.59.3.745 PMC224738 16591620

[55] Leonard TJ, Dick S. Induction of Haploid Fruiting by Mechanical Injury in *Schizophyllum commune*. Mycologia. 1973;65(4):809–22.

[56] Leslie JF, Leonard TJ. Three Independent Genetic Systems that Control Initiation of a Fungal Fruiting Body. Molecular and General Genetics. 1979;171(3):257–60.

[57] Raper JR, Krongelb GS. Genetic and Environmental Aspects of Fruiting in *Schizophyllum commune* Fr. Mycologia. 1958;50(5):707–40.

